# Characterization and expression of fungal defensin in *Escherichia coli* and its antifungal mechanism by RNA-seq analysis

**DOI:** 10.3389/fmicb.2023.1172257

**Published:** 2023-06-14

**Authors:** Yu-Pei Chen, Yingying Li, Fangfang Chen, Hongtan Wu, Shudi Zhang

**Affiliations:** ^1^Department of Public Health and Medical Technology, Xiamen Medical College, Xiamen, Fujian, China; ^2^Engineering Research Center of Natural Cosmeceuticals College of Fujian Province, Xiamen Medical College, Xiamen, Fujian, China; ^3^School of Public Health, Fujian Medical University, Fuzhou, Fujian, China; ^4^Department of Medical Technology, Xiamen Medical College, Xiamen, Fujian, China

**Keywords:** invasive fungal infections, fungal defensin, antifungal protein, *Paecilomyces variotii*, RNA-seq

## Abstract

**Introduction:**

Invasive fungal infections (IFIs) are fatally threatening to critical patients. The fungal defensin as an antifungal protein can widely inhibit fungi.

**Methods:**

In this study, eight antifungal genes from different filamentous fungi were optimized by synonymous codon bias and heterologously expressed in *Escherichia coli*.

**Results and discussion:**

Only the antifungal protein (AFP) from *Aspergillus giganteus* was produced, whereas the AFP from its mutation of the chitin-binding domain could not be expressed, thereby suggesting the importance of the motif for protein folding. In addition, the recombinant AFP (rAFP, 100 μg/mL) pre-heated at 50°C for 1 h effectively inhibited *Paecilomyces variotii* CICC40716 of IFIs by 55%, and no cell cytotoxicity was observed in RAW264.7 cells. After being pre-heated at 50°C for 8 h, the fluorescence emission intensity of the rAFP decreased and shifted from 343 nm to 335 nm. Moreover, the helix and β-turn of the rAFP gradually decreased with the pre-heated treatment temperature of 50°C via circular dichroism spectroscopy. Propidium iodide staining revealed that the rAFP could cause damage to the cell membrane. Moreover, the corresponding differentially expressed genes (DEGs) for downregulation such as amino sugar and nucleotide sugar metabolism, as well as mitogen-activated protein kinase (MAPK) signaling pathway involved in the cell wall integrity were found via the RNA-seq of rAFP treatment. By contrast, the upregulated DEGs were enriched in response to the oxidative stress of Biological Process by the Gene Ontology (GO) database. The encoding proteins of laccase, multicopper oxidase, and nitroreductase that contributed to reactive oxygen species (ROS) scavenging could be recognized. These results suggested that the rAFP may affect the integrity of the cell wall and cell membrane, and promote the increase in ROS, thereby resulting in fungal death. Consequently, drug development could be based on the inhibitory effect of the rAFP on IFIs.

## 1. Introduction

Invasive fungal infections (IFIs) have been increasing because of weak human immunity caused by diseases and the increase in antifungal resistance (Sanguinetti et al., 2019). Thus, they can affect various organs and cause infections such as meningitis, sinusitis, pneumonia, osteomyelitis, and enteritis. IFIs are caused by the filamentous fungus *Aspergillus* and yeast *Candida*. However, with the aid and development of diagnostic technology, non-*Aspergillus* hyaline molds such as *Paecilomyces* spp., *Penicillium* spp., *Fusarium* spp., *Trichoderma* spp., and *Scedosporium* spp. were identified (Jacobs et al., 2020). In general, *Paecilomyces variotii* is distributed in air, soil, water, foods, and decaying plants. Recently, various cases of IFIs caused by *P. variotii* have been successively reported, such as pulmonary mycetoma, cutaneous mycetoma, intravascular infection, and pneumonia (Paixão Marques et al., 2019; Lazarus et al., 2020; Asif et al., 2021; Criado et al., 2021). Fluconazole is commonly used in patients with hematological malignancies, and it can effectively reduce the incidence of IFIs. However, fluconazole cannot protect an individual against *Aspergillus* and non-*Aspergillus* infections (Lionakis et al., 2018). In contrast, voriconazole with an extended spectrum inhibits *Aspergillus* and non-*Aspergillus* fungi (Ben-Ami, 2023). Thus, it has been applied in the clinical treatment of *P. variotii* infection (Sprute et al., 2021). Five patients were successfully cured out of the six cases of *P. variotii* infection.

Defensins derived from fungi have antifungal characteristics and can be found in different molds such as AnAFP, AcAFP, and AFP of *Aspergillus*; PgAFP, PAF, PAFB, and NAF of *Penicillium*; NFAP of *Neosartorya*; FgPAP of *Fusarium*; and MAFP1 of *Monascus* (Tu et al., 2016; Patino et al., 2018; Varadi et al., 2018). The structure of fungal defensin has been explored based on the protein database (PDB) as AFP of *Aspergillus giganteus*, PAFP of *Penicillium chrysogenum*, and NFAP of *Neosartorya fischeri* (Campos-Olivas et al., 1995; Fizil et al., 2015; Huber et al., 2018). Typical fungal defensins contain antiparallel β-sheets with a β-barrel topology and disulfide bonds formed by 6–8 cysteines which may help maintain stability. Moreover, the signal secretion sequences and cutting site of mature protein were observed in the fungal defensins. In addition to being able to resist fungi, the defensin of PAF from *P. chrysogenum* is involved in its apoptotic and autophagic processes (Kovacs et al., 2014). A similar result was also consistent with the AnAFP from *Aspergillus niger* via transcriptome meta-analysis (Paege et al., 2016).

Generally, the antifungal mechanism of defensins is the interaction between the positively charged residues and the negatively charged residues of the plasma membrane, thereby resulting in temporary pores in the cell membrane. This phenomenon can be demonstrated by molecular dynamic simulations and nuclear magnetic resonance (NMR) spectroscopy analyses (Utesch et al., 2018). The γ-core motif of the structurally conserved AFP is involved in protein–membrane interactions, and it indirectly destroys the membranes' integrity through a multi-step process. The defensin enters the fungus, causing an intracellular disturbance, increasing reactive oxygen species (ROS), and leading to fungal death (Oshiro et al., 2019). In addition, the AFP of *A. giganteus* can inhibit chitin synthesis in sensitive fungi (Hagen et al., 2007). Furthermore, a well-conserved bacterial type 3 chitin-binding domain was found in the AFP of *A. giganteus*. The cell wall was further interfered with, and polar growth was retarded. However, the antifungal mechanism of defensin remains unclear.

In the present study, eight antifungal genes derived from different fungi using their optimal codon were heterologously expressed in *Escherichia coli* to produce and characterize fungal defensins. However, only the AFP from *A. giganteus* (CAA43181) was produced. The chitin-binding domain of the AFP was further explored by point mutation assay to verify its importance. *P. variotii* CICC40716 was used for pathogen antagonistic assay. Antifungal capacity, cell cytotoxicity, and thermostability of recombinant AFP were carried out by the Oxford plate assay, propidium iodide (PI) staining, the 3-(4,5-cimethylthiazol-2-yl)-2,5-diphenyl tetrazolium bromide (MTT) assay, fluorescence assay, and circular dichroism spectroscopy. Finally, to examine the difference in gene expression in *P. variotii* CICC40716 with and without the recombinant AFP treatment, an RNA-seq analysis was carried out.

## 2. Materials and methods

### 2.1. Antifungal gene design

The OptimumGene software (GenScript Biotech Corp., Nanjing, China) was used to design eight antifungal genes from *Fusarium poae* (CAR79017), *N. fischeri* (CAQ42994), *A. giganteus* (CAA43181), *Fusarium asiaticum* (CAR79023), *Aspergillus clavatus* (ABR10398), *Colletotrichum gloeosporioides* (ELA33717), *P. chrysogenum* (DOEXD3), and *Fusarium boothii* (CAR79010) for codon optimization analysis (Supplementary Table 1). The optimal sequences were synthesized and introduced into pET-23a(+) within the *Sal*I/*Xho*I sites by GeneDireX Inc. (Las Vegas, NV, United States) for the expression of plasmids to be obtained.

### 2.2. Expression of antifungal protein in *E. coli*

For the confirmation of the expression of antifungal proteins, these plasmids were transformed into *E. coli* C43(DE3) to confirm. A single colony of transformants was obtained from a Luria–Bertani (LB) agar plate with 100 μg/ml of ampicillin. The transformant was incubated overnight in an LB broth and then transferred into a fresh LB broth at 37°C until OD_600_ reached 0.4–0.5. The culture was incubated with and without IPTG (0.5 mM) at 25°C overnight. Afterward, the cells disrupted by a JY92-IIN sonicator (Ningbo Scientz Biotechnology, Ningbo, China) were harvested and suspended in a native buffer (50 mM NaH_2_PO_4_, pH 8.0, and 0.5 M NaCl). Subsequently, the supernatant was verified by sodium dodecyl sulfate-polyacrylamide gel electrophoresis (SDS-PAGE) and Coomassie blue staining. Furthermore, a Western blot analysis was carried out as described by Sambrook and Russell (2001). A primary antibody against His-tag at a dilution of 1:1,000 (BBI Life Sciences, Shanghai, China) and horseradish peroxidase-conjugated antibody to mouse immunoglobulin G (1:5,000 dilution; West Grove, PA, United States) were utilized. Finally, proteins were identified and performed using a SuperSignal West Pico Kit (Thermo Fisher Scientific Pierce, IL, United States).

### 2.3. Purification of antifungal protein

The antifungal protein was purified by a Ni-NTA purification system (Invitrogen, Carlsbad, CA, United States) and dissolved in a native buffer. The dissolved protein was dialyzed against the 1 × native buffer, and the buffer was replaced four times for 2 days, followed by 0.1 × native buffer for 1 day to remove the imidazole from the native elution buffer (Ni-NTA purification system). The dialysis protein was concentrated by lyophilization for antifungal analysis. The purified antifungal protein from *A. giganteus* (CAA43181) was designated as the rAFP.

### 2.4. Point mutation of antifungal protein

The point mutation of the chitin-binding domain was designed to verify the chitin-binding domain of recombinant AFP from *A. giganteus* (CAA43181) as follows: *R*YKAQ: *A*GGTACAAAGCGCAG (K15R); KNKAQ: AAGAACAAAGCGCAG (Y16N); KYRAQ: AAGTACAGAGCGCAG (K17R); KYKVQ: AAGTACAAAGTGCAG (A18V); KYKAT: AAGTACAAAGCGACG (Q19T). The AFP genes with different chitin-binding domains were synthesized and introduced into pET-23a(+) within the *Sal*I/*Xho*I sites by Azenta Life Sciences (Suzhou, Jiangsu, China) to obtain the expression plasmids. These plasmids were expressed in *E. coli* C43(DE3) and confirmed by the SDS-PAGE and Western blot analysis.

### 2.5. Antifungal assay of the rAFP

The Oxford plate assay was used to determine the antagonistic dosage of anti-fungi. The *P. variotii* CICC40716 was cultivated in Petri dishes containing potato dextrose agar. The Oxford plates with different concentrations of the rAFP from 0 to 200 μg were set on the plate with *P. variotii* CICC40716 for 30°C cultivation. The clear zone of fungal inhibition was observed after 24 h of culture. In addition, the *P. variotii* CICC40716 spores obtained from the PDA plate were introduced into the PDB medium with OD_600_ of ~0.1–0.2 about 3 × 10^6^ spores/mL to 8 × 10^6^ spores/mL. Then, 1 ml of broth with spores was added to a 24-well plate. Different concentrations of the rAFP (0, 12.5, 25, 50, and 100 μg/ml) pre-treated from 30 to 80°C for 1 h were prepared to determine the antifungal rate by an enzyme-linked immunosorbent assay (ELISA) reader (Molecular Devices, Sunnyvale, CA, United States) at 600 nm. Furthermore, 100 μg/ml of rAFP was incubated at 50°C for 1–8 h to explore the thermostability of the rAFP. The rAFP of thermal treatment was immediately chilled and used for antifungal rate assay in a 24-well plate.

### 2.6. Effect of the rAFP on cell viability

The cell viability of the RAW264.7 cell was determined by the MTT method to verify the cytotoxicity of the rAFP. The RAW264.7 cells were placed into a 96-well plate for 24 h and incubated at 5% CO_2_ and 37°C. The rAFP proteins with the final concentrations of 0, 12.5, 25, 50, and 100 μg/ml were added to the RAW264.7 cells for 24 h of reaction. The supernatant was removed, and cells were washed with PBS buffer. The fresh medium with 20 μl of MTT (0.5%) was added to the cells for 4 h of reaction and 5% CO_2_ incubation at 37°C. Finally, the medium was discarded, and 150 μl of DMSO was utilized to dissolve the crystal product. The absorbance of OD_510_ was measured using an ELISA reader (Molecular Devices).

### 2.7. Fluorescence assay of the rAFP

The rAFP of thermal treatment with a final concentration of 100 μg/ml was utilized for fluorescence assay by an ELISA reader (Molecular Devices). The wavelength range of 300–500 nm with excitation at 280 nm was performed. The slit width of 4.66 nm was set in both excitation and emission.

### 2.8. Protein secondary structure assay of the rAFP

The secondary structure of 100 μg/ml of rAFP with thermal treatment was analyzed over the wavelength of 180–260 nm by circular dichroism spectroscopy (Applied Photophysics, UK). The bandwidth and time-per-point were set to 1.0 nm and 0.5 s, respectively. The wavelength was recorded and treated by subtracting the baseline, and the CDNN software was utilized to evaluate the secondary structure content of the protein.

### 2.9. PI staining of fungi

The *P. variotii* CICC40716 spores were prepared with an OD_600_ of 0.1–0.2 and mixed with the rAFP (0, 50, and 100 μg/ml) at 30°C for 24 h of incubation. The hyphae were harvested and washed with a PBS buffer three times. The hyphae were suspended in a PBS buffer, and 20 μl of PI (100 μg/ml) was added for 5 min of reaction in the dark. The fluorescence image was obtained with a fluorescence microscope (Olympus BX43F-R, Tokyo, Japan).

### 2.10. RNA-seq analysis of *P. variotii* CICC40716

The *P. variotii* CICC40716 spores were introduced into fresh potato dextrose broth with an OD_600_ of 0.1–0.2 in the presence and absence of the rAFP (50 μg/ml). The *P. variotii* CICC40716 broth was cultured at 30°C for 24 h. *P. variotii* CICC40716 was harvested by Biomarker Technologies (Beijing, China) for RNA extraction, cDNA synthesis, library construction, cDNA sequencing, and *de novo* analysis. cDNA sequencing was carried out by an Illumina NovaSeq 6000 (Illumina, San Diego, CA). The sequencing reads were assembled and aligned by Trinity (Grabherr et al., 2011) and bowtie2 (Langmead et al., 2009). The unigenes were subjected to DIAMOND (Buchfink et al., 2015) for NR (Deng et al., 2006), Swiss-Prot (Apweiler et al., 2004), COG (Tatusov et al., 2000), and KEGG (Koonin et al., 2004) analyses. The InterProScan (Jones et al., 2014) was performed to obtain the result of GO Orthology (Ashburner et al., 2000). The differentially expressed genes (DEGs) were compared using DESeq2 (Love et al., 2014). A false discovery rate of < 0.01 and a fold change of >2 were used as the criteria. These RNA-seq data were deposited at DDBJ/ENA/GenBank under the accession no. of BioProject PRJNA924260, and SRA, SRR23091293, SRR23092107, SRR23095547, SRR23095642, SRR23095643, and SRR23095641.

### 2.11. Quantitation of gene expression by real-time RT-PCR

RNA was obtained according to the experimental condition of the RNA-seq analysis to confirm the result of RNA-seq. The HiFi-MMLV cDNA kit with oligo (dT) and random hexamers (Beijing ComWin Biotech, China) was utilized for the synthesis of first-strand cDNA at 42°C for 1 h of reaction. The cDNA was mixed with the UltraSYBR Mixture (Beijing ComWin Biotech) in a final volume of 12.5 μl for real-time PCR using the Roche LightCycler^®^ 480 System (Roche Group, Switzerland). The thermal cycling parameters included initial heating at 95°C for 10 min, denaturing at 95°C for 10 s, annealing at 58°C for 30 s, and extension at 72°C for 32 s, with an amplification of 40 cycles. The primer set of 18S rRNA as the internal control was 18SrRNA-F: CGATGGAAGTTTGAGGCAAT and 18SrRNA-R: CACGACGGAGTTTCACAAGA. The primer sets of *P. variotii* CICC40716 genes were C24077-F: GCGTCGTCGCAATGTACTTC, C24077-R: CCAGACCACGACTGTCAAGT; C24160-F: CCACCAGCAGTTATCAAGGG, C24160-R: GCCAGAACTATGACAGGCAC; C24458-F: TCCCTGGGTCTGCTCAACAA, C24458-R: GTCATCAACTCAGGACGACC; C21938-F: AGGCTTCAACAAGCTCTCCG, C21938-R: CTCGCTGCCATGTTCGGAAT; C20865-F:GGGGAAGGGGGTCGTTTAAA, C20865-R:CCTAACCCGGACAGTCCTTT; C22577-F:GCCAAGGAGAGGTGACAATG, C22577-R:GGAGCTTATTCGAAGCTGGC; C13492-F:CTTTTCAGGGTCCTGGTACG, C13492-R:GCTGCTCATTACTCGTCCTC; and C13322-F:TCTTCGAGGTCGTCTGGCAA, C13322-R:GAGTACAGGTATGCAGCTGG.

## 3. Results

### 3.1. Expression and purification of antifungal protein

**Figure 1:**
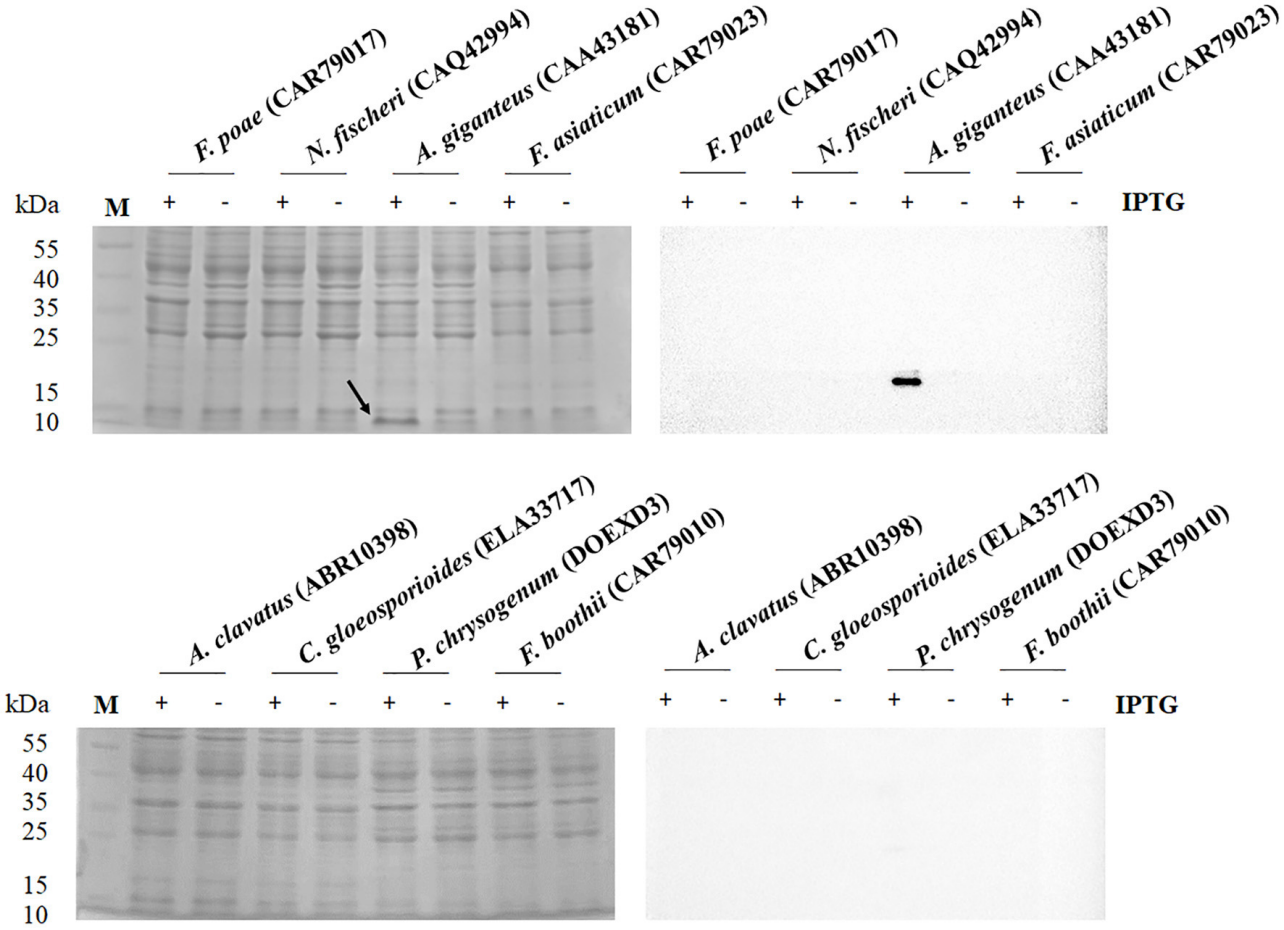
SDS-PAGE **(left)** and Western blot **(right)** analysis of antifungal protein from the protein accession numbers of CAR79017, CAQ42994, CAA43181, CAR79023, ABR10398, ELA33717, DOEXD3, and CAR79010. The antifungal proteins were carried out in the presence (+) and absence (–) of IPTG at 0.5 mM. The arrow indicates the expressed protein with an expected molecular mass of 9.2 kDa. The M indicates the protein marker.

OptimumGene for heterologous expression of *E. coli* was used to optimize the codons of eight antifungal genes from *F. poae* (CAR79017), *N. fischeri* (CAQ42994), *A. giganteus* (CAA43181), *F. asiaticum* (CAR79023), *A. clavatus* (ABR10398), *C. gloeosporioides* (ELA33717), *P. chrysogenum* (DOEXD3), and *F. boothii* (CAR79010). The optimal antifungal sequences were synthesized and cloned into pET-23a(+) by the restriction sites of *Sal*I and *Xho*I, with C-terminal His-tag triggered by the T7 promoter. The expression plasmids were introduced into *E. coli* C43(DE3). The band with an approximate molecular mass of 9.2 kDa derived from *A. giganteus* (CAA43181) could be observed on the SDS-PAGE after gene expression with IPTG induction in *E. coli* C43 (DE3) (Figure 1). The recombinant AFP fused with His-tag was verified through a Western blot analysis. The recombinant AFP was purified using a nickel column, and a major band on SDS-PAGE was observed (Supplementary Figure 1). After purification, the yield of the recombinant AFP was ~780 μg/L of culture. The purified recombinant AFP, namely, rAFP, was further used for follow-up analyses of antifungal activity, cell viability, thermostability, and RNA-seq.

### 3.2. Chitin-binding domain assay of the antifungal protein

**Figure 2:**
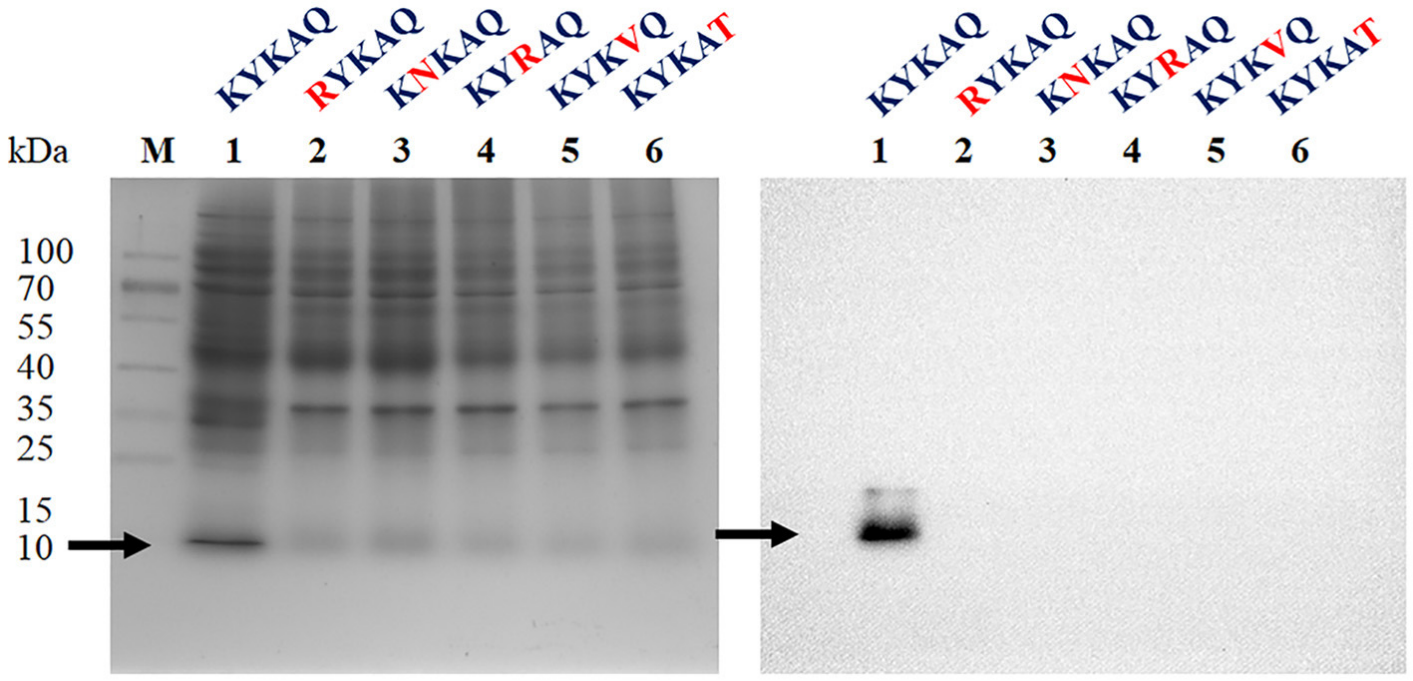
SDS-PAGE **(left)** and Western blot **(right)** analysis of rAFP with five-point mutations of the chitin-binding motif (KYKAQ). The red letter indicates the point mutations of the chitin-binding motif. The arrow indicates the expressed protein with the expected molecular mass of 9.2 kDa. The M indicates the protein marker.

Herein, the CKYKAQ sequence of the AFP from *A. giganteus*, which was similar to a conserved motif of AKWWTQ in the bacterial type 3 chitin-binding domain, was observed (Hagen et al., 2007). The point mutation of the antifungal protein was performed via sequence synthesis, which was introduced into the expression vector pET-23a(+) of *E. coli* to confirm the role of the chitin-binding domain in antifungal activity. Each point mutation of KYKAQ was replaced based on its related amino acid characteristics such as RNRVT. Five expression plasmids containing K15R, Y16N, K17R, A18V, and Q19T were cloned and expressed in *E. coli*. However, no protein was expressed at each point mutation based on the SDS-PAGE gel and Western blot analysis (Figure 2).

### 3.3. Antifungal ability of the rAFP on *P. variotii* CICC40716

**Figure 3:**
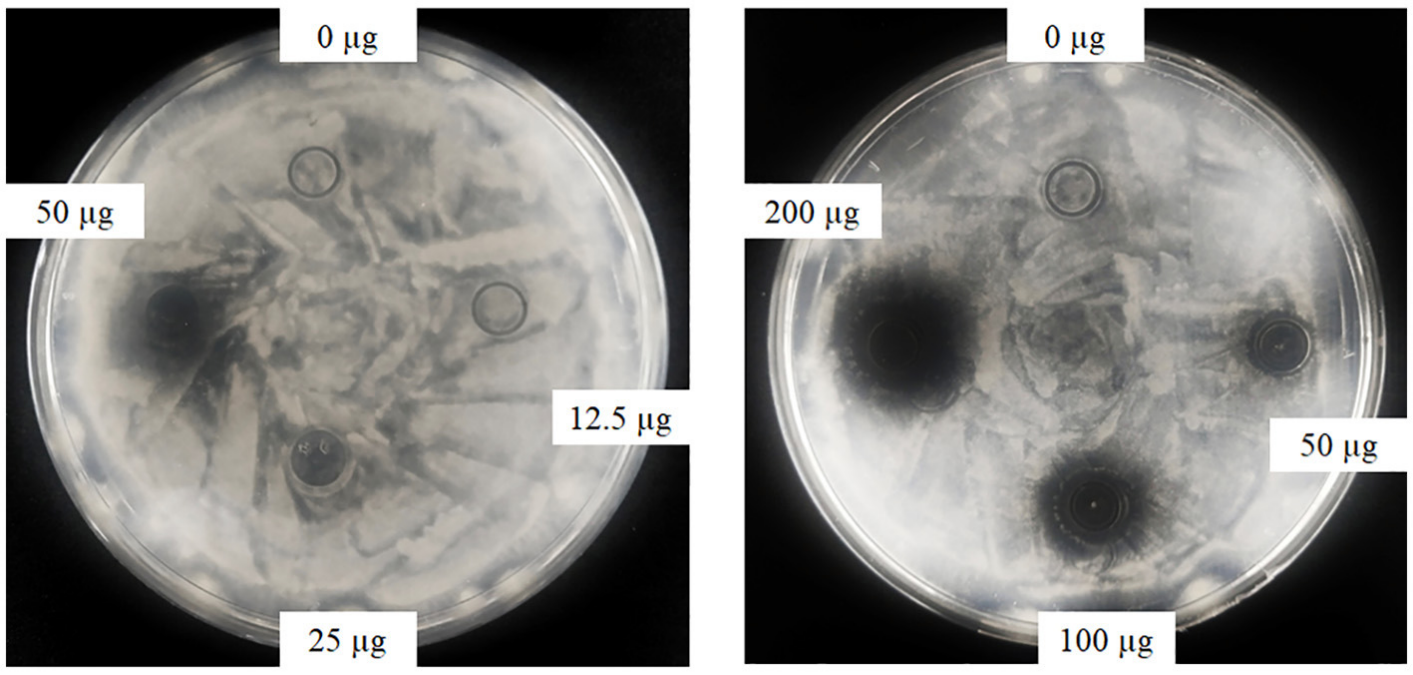
Antifungal activity of the rAFP with different concentrations (0, 12.5, 25, 50, 100, and 200 μg) against *P. variotii* CICC40716 using the Oxford plate assay. The plates were cultured at 30°C for 24 h, while the growth inhibitions of *P. variotii* CICC40716 were observed.

**Figure 4:**
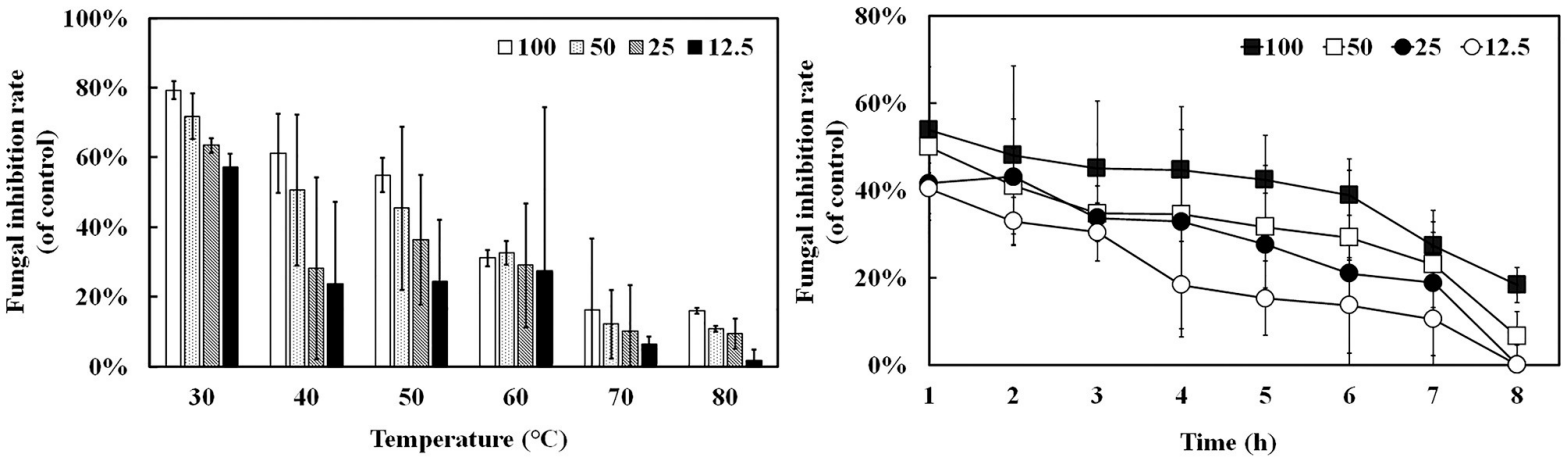
Antifungal activity of the rAFP pre-heated from 30 to 80°C for 1 h with different concentrations (12.5, 25, 50, and 100 μg/mL) against *P. variotii* CICC40716. The thermostability assay of the rAFP pre-heated at 50°C from 1 to 8 h. The fungal inhibition rate was calculated as follows: [(OD_600_ of control-OD_600_ of rAFP treatment)/OD_600_ of control] × 100.

Oxford plate assay and antifungal rate assay were conducted to verify the antifungal activity of purified rAFP against *P. variotii* CICC40716. The Oxford plate assay revealed an antifungal clear zone by adding 50 μg of rAFP (Figure 3). As the concentration of rAFP increased, the clear zone was discernible. In addition, different doses of rAFP were pre-heated from 3 to 80°C for 1 h, which were utilized for the antifungal rate assay in 1 ml of broth of a 24-well plate. The result showed that a 79.3% inhibition rate of *P. variotii* CICC40716 was attained after 100 μg/ml of rAFP pretreatment at 30°C for 1 h (Figure 4). Even if the rAFP was as low as 12.5 μg/ml, the fungal inhibition rate remained at 57.3%. As the temperature increased, the antifungal rate declined. The fungal inhibition rate was lower than 20% after 100 μg/ml of rAFP was pretreated at 70°C for 1 h. In contrast, the rAFP pre-heated at 50°C from 1 to 8 h was used to measure its fungal inhibition rate to detect the thermostability of antifungal protein. After 7 h of thermal pretreatment, the antifungal rate of 100 μg/ml rAFP reduced to half of that after 1 h. However, no fungal inhibition was observed when the rAFP was lower than 25 μg/ml after 8 h of thermal pretreatment.

### 3.4. Cell viability under the rAFP treatment

**Figure 5:**
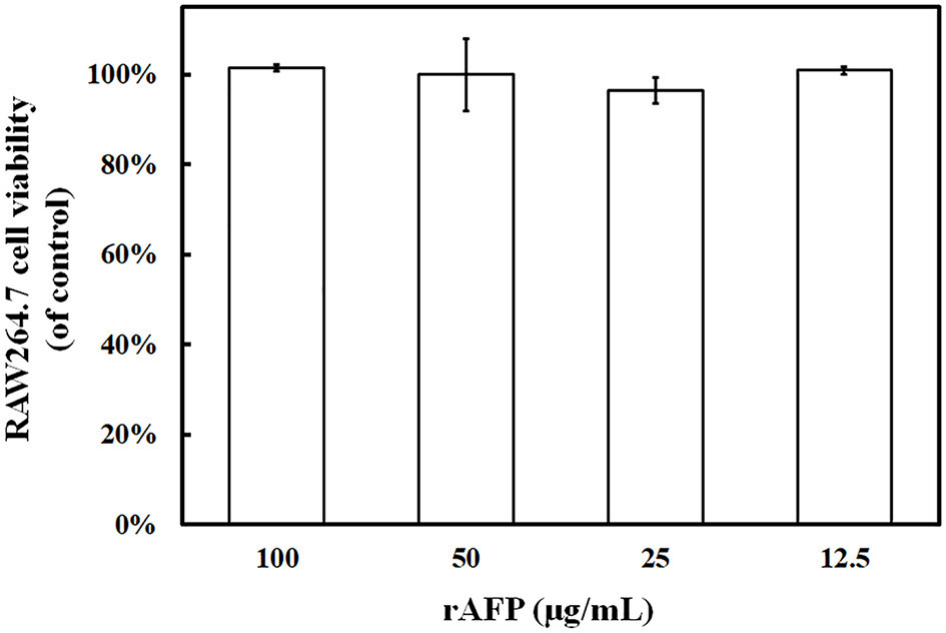
RAW264.7 cell viability treated with the rAFP of different concentrations (12.5, 25, 50, and 100 μg/ml). The cell viability was calculated as follows: (OD_510_ of rAFP treatment/OD_510_ of control) × 100.

The RAW264.7 cell was utilized to detect the effect of rAFP on cell viability to identify the potential of rAFP as a medical treatment. Given that 100 μg/ml of rAFP can achieve a good fungal inhibition rate, the effect of different doses of rAFP from 0 to 100 μg/ml on RAW264.7 cells was analyzed. No cytotoxicity was observed, and the survival rate of the RAW264.7 cells was maintained at almost 100% (Figure 5).

### 3.5. Effect of temperature on the rAFP protein structure by fluorescence and circular dichroism spectroscopy

**Figure 6:**
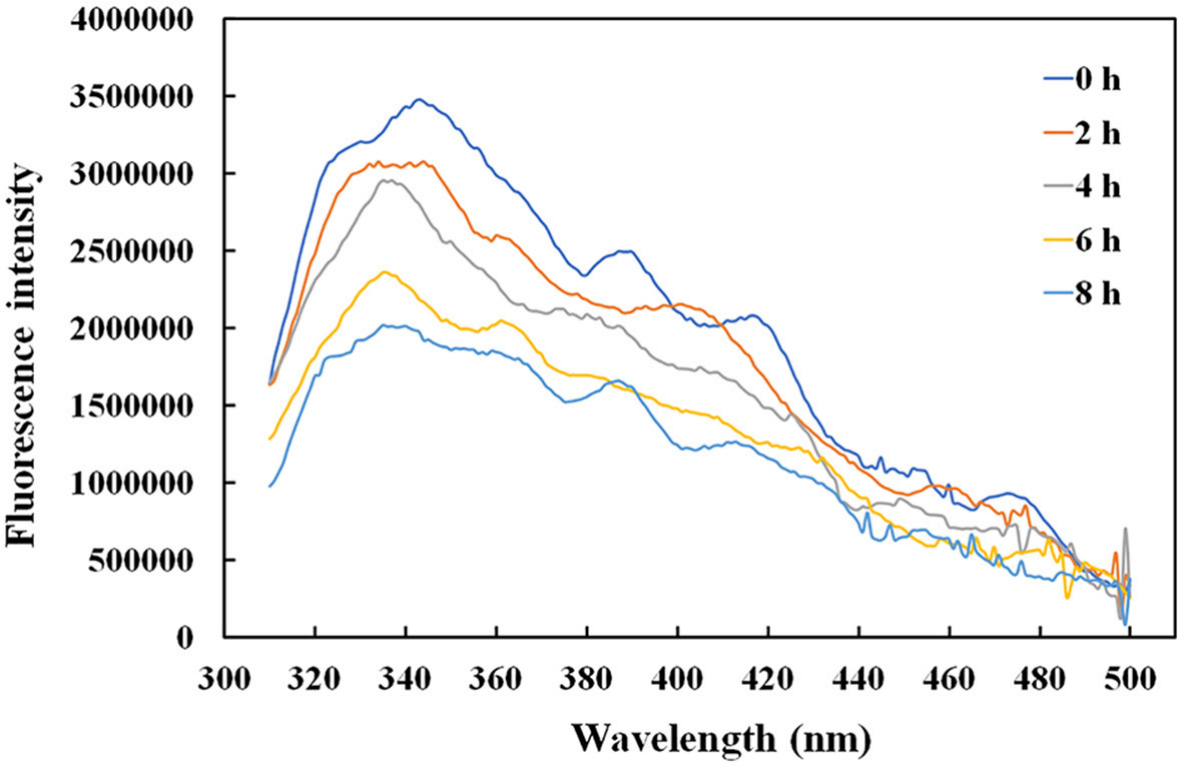
Fluorescence spectra of rAFP pre-heated at 50°C from 0 to 8 h using an ELISA reader.

Given that the intrinsic fluorophores of tyrosine and phenylalanine occupying 10% of the rAFP with six Tyr and one Phe were distributed around the cysteine disulfide bonds, the effect of temperature on the antifungal protein structure was investigated using the fluorescence spectroscopy. The rAFP had a strong emission at 343 nm under an excitation of 280 nm. The change in fluorescence emission intensity was observed in the rAFP pre-heated at 50°C from 0 to 8 h (Figure 6). The fluorescence emission intensity of the rAFP decreased and shifted to 335 nm with an increase in thermal pretreatment. After the thermal pretreatment for 2, 4, 6, and 8 h, the fluorescence emission intensity of the rAFP was reduced by 12.0, 19.4, 37.2, and 43.2%, respectively, based on the strong emission at 343 nm.

Thus, CD spectroscopy in the wavelength range of 180–260 nm was performed to further study the change in the protein structure of rAFP by thermostability (Table 1). The results indicated that the amount of helix and β-turn in the rAFP decreased with the increase in thermal pretreatment. After pre-heating for 8 h at 50°C, the helix and β-turn of the rAFP were reduced by 47 and 33.9%, respectively.

**Table 1:** CD spectra of rAFP pre-heated at 50°C from 0 to 8 h.

**Time**	**Helix**	**Antiparallel**	**Parallel**	**β-Turn**	**Random coil**
0 h	21 ± 0.4%	70.7 ± 2.2%	13.8 ± 1.7%	24.7 ± 0.6%	38.1 ± 1.1%
1 h	20.77 ± 0.61%	41.77 ± 3.21%	16.3 ± 0.3%	21.13 ± 0.57%	46.07 ± 0.32%
2 h	18.57 ± 0.85%	28.17 ± 2.25%	14.17 ± 0.59%	19.8 ± 0.2%	45.47 ± 0.35%
3 h	15.87 ± 0.9%	26.4 ± 3.46%	14.37 ± 0.9%	18.67 ± 0.4%	43.8 ± 0.35%
4 h	14.83 ± 0.35%	40.67 ± 0.58%	14.23 ± 1.32%	18.4 ± 0.26%	42.73 ± 0.78%
5 h	13.6 ± 0.35%	40.27 ± 0.58%	18.13 ± 1.01%	17 ± 0.62%	59.23 ± 1.18%
6 h	12.8 ± 0.3%	42.8 ± 0.46%	15.27 ± 1.04%	17.43 ± 0.45%	41.03 ± 0.51%
7 h	12.1 ± 0.3%	38.93 ± 0.64%	21.17 ± 0.75%	16.73 ± 0.4%	57.37 ± 0.67%
8 h	11.13 ± 0.68%	41.73 ± 2.35%	16.8 ± 0.61%	16.33 ± 0.55%	44.8 ± 0.85%

### 3.6. Effect of the rAFP on the cell membrane integrity of *P. variotii* CICC40716

**Figure 7:**
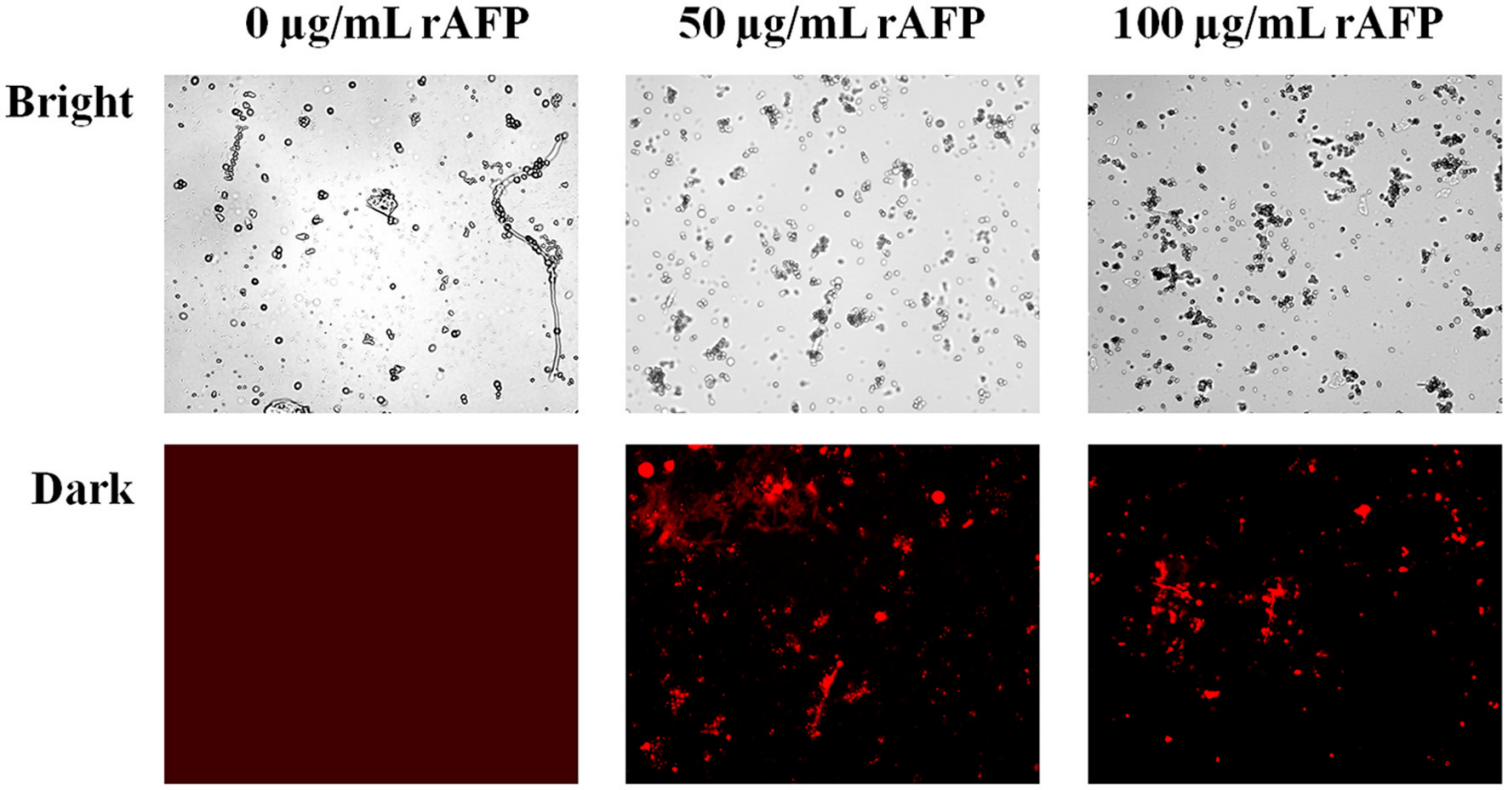
Morphology (bright field) and the corresponding PI staining (dark field) of *P. variotii* CICC40716 treated with the rAFP of different concentrations (0, 50, and 100 μg/ml).

To confirm whether the rAFP will affect the integrity of the cell membrane, PI, a fluorescent dye, was utilized. PI dye can enter the damaged cell membrane and react with DNA and RNA to emit red fluorescence. Fluorescence microscope observations were made in the presence and absence of the rAFP for the *P. variotii* CICC40716. However, no red fluorescence was observed in the control, thereby indicating the intact cell membrane of *P. variotii* CICC40716 (Figure 7). The red fluorescence in *P. variotii* CICC40716 was significantly perceived in the presence of 50 and 100 μg/ml of rAFP. Thus, the cell membranes of the *P. variotii* CICC40716 were damaged, and their permeability was improved.

### 3.7. RNA-seq analysis of *P. variotii* CICC40716 with the rAFP treatment

For the RNA-seq analysis, *P. variotii* CICC40716 was treated with and without rAFP (50 μg/ml). An average of 21,982,300 clean reads was obtained from each treatment in triplicate (Supplementary Table 2). The clean reads of each sample were mapped back to the assembled transcripts. The assembled transcripts accounted for an average of 92.46% of clean reads. Pearson's correlation was carried out to examine the gene expression correlation of the triplicates. The results showed that the triplicate of each treatment with and without the rAFP had a high correlation with an average of 0.99 and 0.93, respectively.

**Figure 8:**
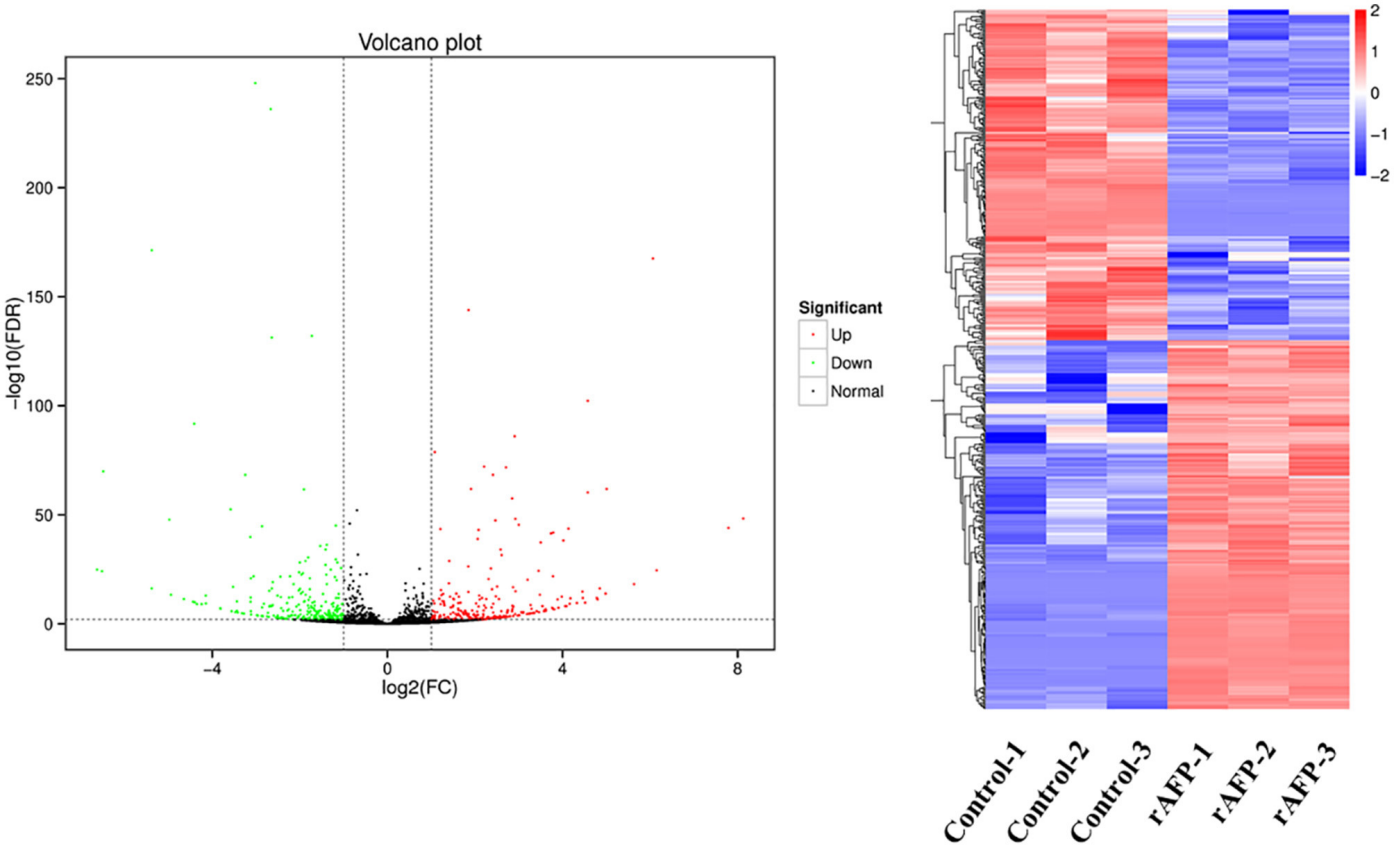
Volcano plots and the cluster of the DEGs by *P. variotii* CICC40716 treated with and without the rAFP. The red and green dots indicated significant DEGs with upregulation and downregulation, respectively, in the volcano assay. Control-1, Control-2, and Control-3 indicate the DEGs' cluster of *P. variotii* CICC40716 in triplicate without the rAFP treatment. rAFP-1, rAFP-2, and rAFP-3 indicate the DEGs cluster of *P. variotii* CICC40716 in triplicate with the 50 μg/mL rAFP treatment.

**Figure 9:**
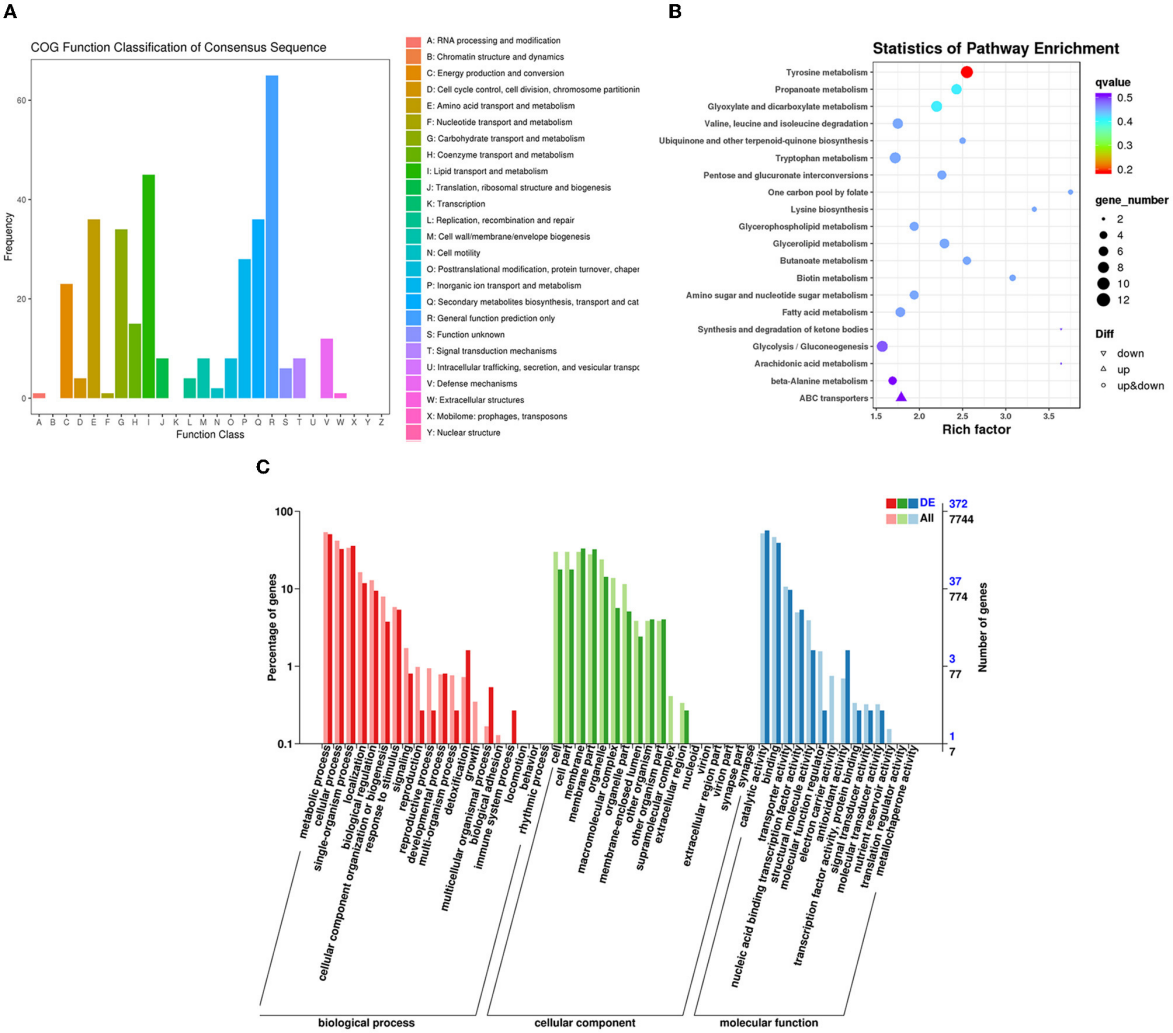
Classification of DEGs by *P. variotii* CICC40716 treated with and without the rAFP via the **(A)** COG, **(B)** KEGG, and **(C)** GO databases.

**Figure 10:**
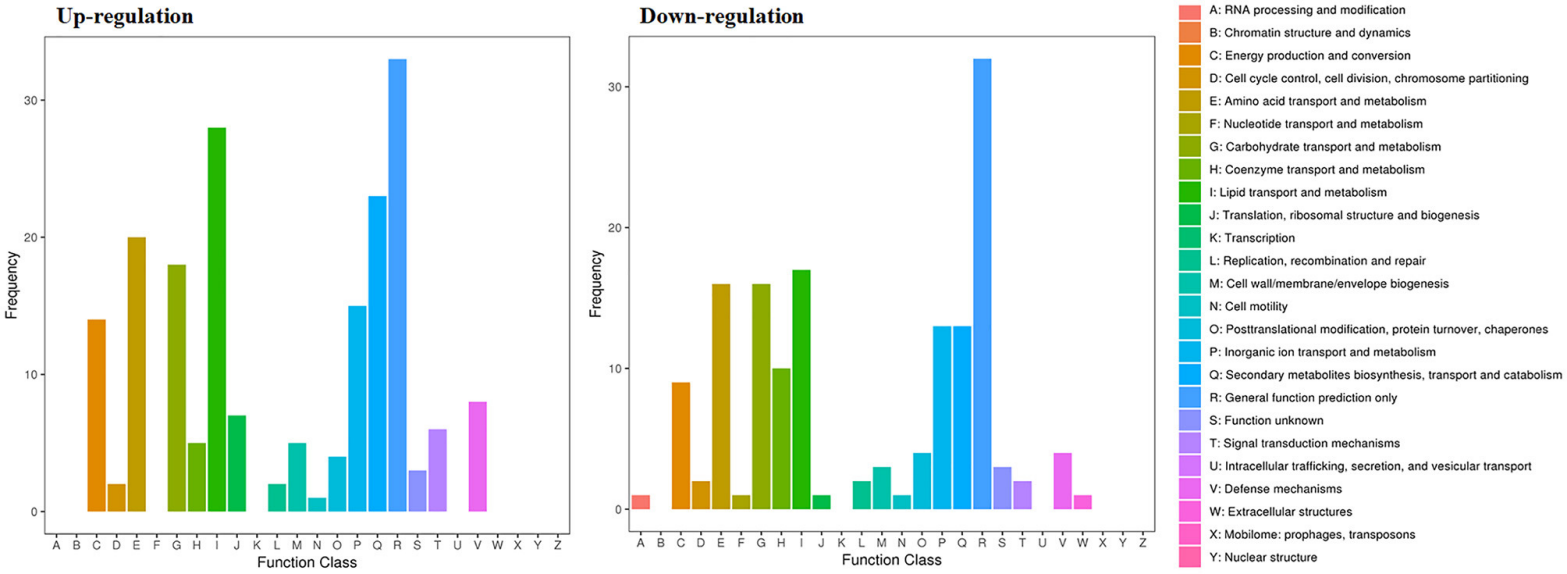
Gene expression of the DEGs by *P. variotii* CICC40716 treated with and without the rAFP via the COG database.

**Figure 11:**
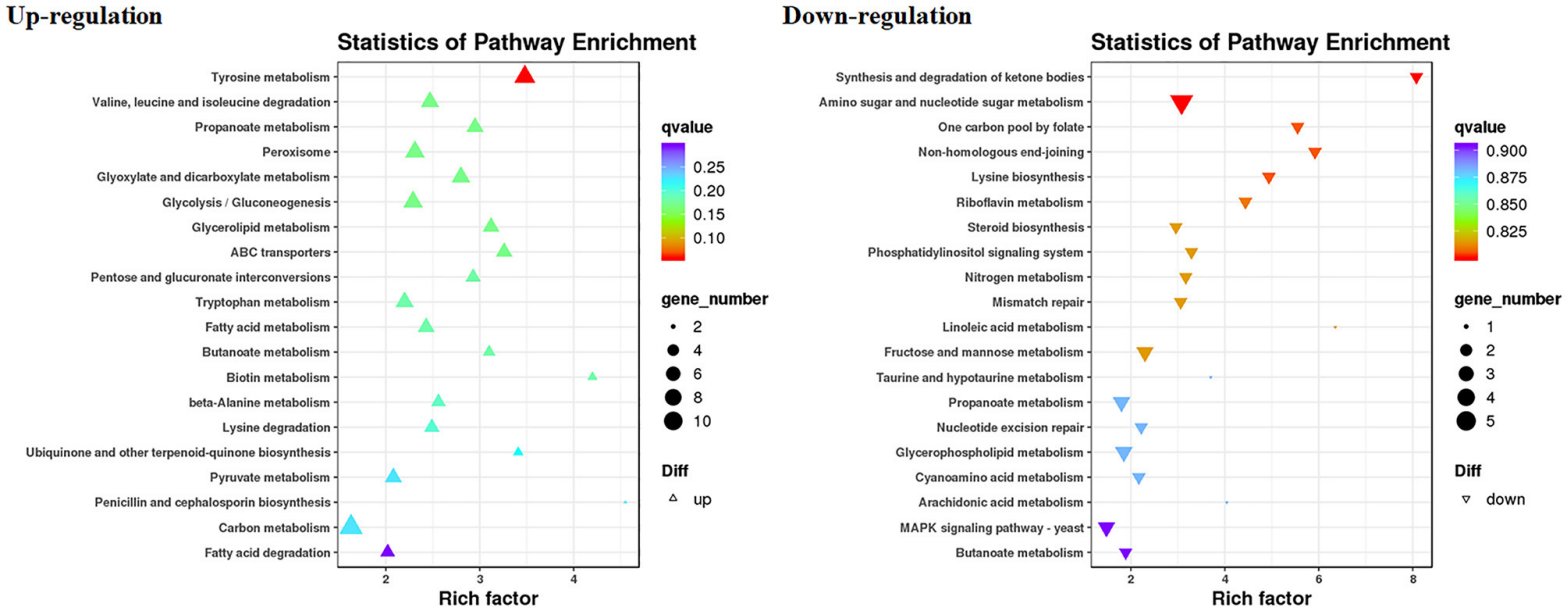
Gene expression of DEGs by *P. variotii* CICC40716 treated with and without the rAFP via the KEGG pathway.

**Figure 12:**
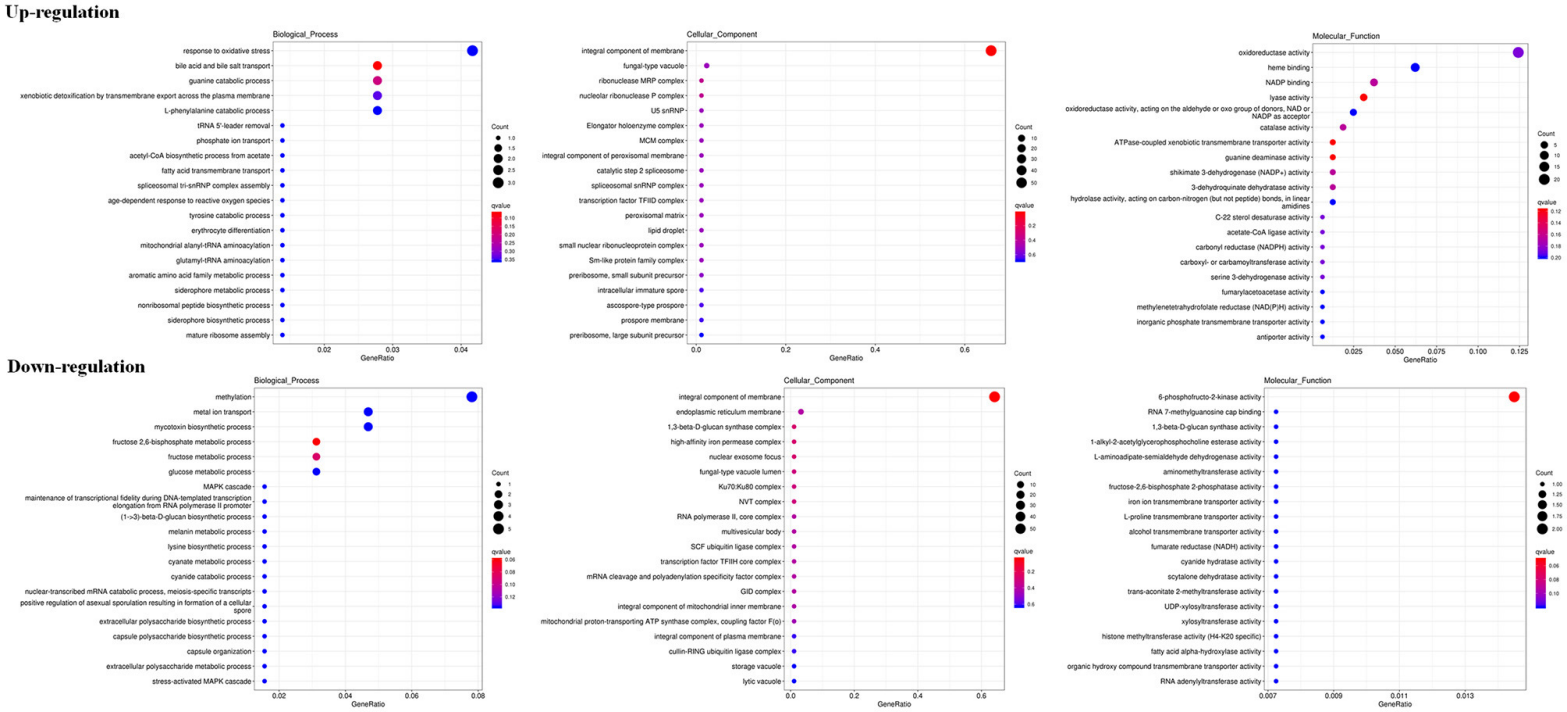
Gene expression of DEGs by *P. variotii* CICC40716 treated with and without the rAFP via the GO database.

The RNA sequencing results suggested that a total of 10,177 unigenes with a threshold of BLAST E-value not larger than 1e-5 and HMMER E-value larger than 1e-10 were detected in these samples. A total of 5,164, 7,744, and 5,983 unigenes were annotated based on the COG, GO, and KEGG databases, respectively. After rAFP treatment, 531 DEGs with more than a 2-fold change in *P. variotii* CICC40716 were identified by the volcano plot (Figure 8). The numbers of upregulated and downregulated genes were 280 and 251 genes, respectively. COG, GO classification, and KEGG pathway of DEGs are shown in Figure 9. The 15 most upregulated and downregulated genes with the rAFP treatment are shown in Supplementary Tables 3, 4. The multidrug transporter genes corresponding to the resistance of antifungal agents can be influenced by antifungal drugs (Wu et al., 2016; Samaras et al., 2020). Table 2 shows the effect of the rAFP on the genes involved in the multidrug transporter. In the COG database, general function prediction only (R), lipid transport and metabolism (I), secondary metabolites biosynthesis, transport and catabolism (Q), amino acid transport and metabolism (E), carbohydrate transport and metabolism (G), and inorganic ion transport and metabolism can be perceived in the most upregulated and downregulated function classes (Figure 10). In the KEGG pathway, the rich factor indicated the ratio of the DEGs (sample) enriched in the pathway to the amounts of annotated genes (background) (Figure 11). Moreover, the closer the *q*-value is to 0, the more significant the enrichment is. Biotin and tyrosine metabolism were observed in the high rich factor and low q-value in the upregulation of the KEGG pathway, respectively, whereas synthesis and degradation of ketone bodies and amino sugar and nucleotide sugar metabolism were found in that of the downregulation of the KEGG pathway. On the one hand, in the GO database, the most upregulated genes involved in response to oxidative stress, integral component membrane, and oxidoreductase activity were shown in biological processes, cellular components, and molecular functions, respectively (Figure 12). On the other hand, the most downregulated genes involved in methylation, integral component membrane, and 6-phosphofructo-2-kinase activity were identified in biological processes, cellular components, and molecular functions, respectively.

**Table 2:** Multidrug resistance transporter genes responding to the ratio of the rAFP treatment to control *P. variotii* CICC40716.

**Regulation**	**ID**	**DNA size (bp)**	**NR annotation**	**Swiss-prot annotation**	**log_2_ ratio**
Upregulation	c13492.graph_c0	3,198	MFS transporter, putative	Major facilitator superfamily multidrug transporter mfsB	5.633
	c20529.graph_c0	2,162	Lactose permease	MFS-type transporter oryC	3.981
	c22476.graph_c0	2,493	Hypothetical protein	Probable glucose transporter HXT5	3.501
	c22033.graph_c0	1,097	Hypothetical protein	MFS-type transporter oryC	2.846
	c20536.graph_c0	5,933	ABC drug exporter AtrF	ABC multidrug transporter atrF	2.751
	c17990.graph_c1	2,262	ABC multidrug transporter Mdr1	ABC multidrug transporter mdr1	2.102
	c16566.graph_c0	2,157	Putative siderophore iron transporter	Major facilitator copper-regulated transporter crmC	2.027
	c17990.graph_c0	3,108	ABC multidrug transporter Mdr1	ABC multidrug transporter mdr1	1.908
	c21886.graph_c0	2,626	Putative MFS drug efflux pump	MFS-type transporter VdtG	1.477
	c13207.graph_c0	2,707	MATE efflux family protein subfamily	Uncharacterized transporter	1.185
	c19500.graph_c0	1,105	MFS transporter	–	1.017
Downregulation	c22950.graph_c0	1,538	MFS general substrate transporter	MFS transporter PfmaC	−3.419
	c19986.graph_c0	2,067	Hypothetical protein PVAR5_0967	Transporter mfs1	−2.820
	c23933.graph_c2	1,908	MFS monosaccharide transporter	Low-affinity glucose transporter HXT3	−2.694
	c22200.graph_c0	9,011	Hypothetical protein	Uncharacterized MFS-type transporter	−1.578
	c23297.graph_c1	1,181	MFS transporter	MFS transporter M2	−1.552
	c18577.graph_c0	2,637	MFS transporter	Uncharacterized membrane protein	−1.178
	c22560.graph_c1	9,533	Major facilitator superfamily domain-containing protein	Multidrug resistance protein fnx1	−1.015
	c22719.graph_c0	1,864	MFS monocarboxylate transporter	MFS-type transporter	−1.010

Eight genes containing four upregulated genes and four downregulated genes were randomly selected for real-time RT-PCR to verify the findings of RNA-seq analysis. The results revealed that the gene regulation using real-time RT-PCR was consistent with the change in RNA-seq with and without the rAFP treatment (Supplementary Table 5).

## 4. Discussion

Some studies have been conducted on the heterologous expression of antifungal proteins. Recombinant mature AFP from *A. giganteus* expressed in the yeast *Pichia pastoris* is indistinguishable from the natural AFP (Lopez-Garcia et al., 2010). However, Glu-Ala-Glu-Ala-Glu repeats at the N-terminal of the AFP inactivated its antifungal activity. Moreover, the AFP was transferred into *A. niger* and had a very low expression level with a maximal concentration of 350 μg/L (Wnendt et al., 1994). Meanwhile, MAFP1 derived from *Monascus pilosus* can be expressed and obtained from *E. coli* with approximately 1 mg/L of culture (Tu et al., 2016). Nevertheless, the recombinant MAFP1 has lower antifungal activity than that of the native protein. Therefore, the heterologous expression of fungal defensin is difficult. Thus, in the present study, the codon optimization of fungal defensin was carried out to avoid the incompatibility of codon usage bias, resulting in low expression or protein misfolding. Unfortunately, only the AFP derived from *A. giganteus* was expressed in *E. coli* (Figure 1). Even if the antifungal protein between *A. giganteus* (CAA43181) and *A. clavatus* (ABR10398) had only two amino acid differences, the usage of synonymous codons for gene expression may be the factor that caused the defensin derived from *A. clavatus* to fail to express in *E. coli*. Furthermore, the His-tag fused on the N-terminus or C-terminus of AFP may result in different expression levels. The C-terminal His-tag may interrupt the protein folding process of the other antifungal proteins (Li et al., 2009). Thus, the recombinant AFP of *A. giganteus* (CAA43181) was used to study its characterization and antifungal mechanism.

A conserved chitin-binding domain, CKYKAQ, involved in the inhibition of chitin synthesis of sensitive fungi existed in AFP of *A. giganteus* (Hagen et al., 2007). K15R, Y16N, K17R, A18V, and Q19T were the five-point mutations designed for heterologous expression in *E. coli* to characterize the chitin-binding domain. Unexpectedly, no protein in these mutations was produced (Figure 2). Cys14 was considered a disulfide bond pairing to Cys40, and Cys14 to Tyr16 was located in one of the five antiparallel β-strands based on the protein structure of AFP in the PDB (Campos-Olivas et al., 1995). In addition, Lys15, Tyr16, and Ala18 had polar contacts with the adjacent amino acids such as Lys6, Tyr8, Thr23, and Gly21, respectively, based on a PyMOL analysis (Supplementary Figure 2) (Janson et al., 2017). Although no polar contact was found in Lys17 and Gln19, Gly21 and Tyr3 were close to the two amino acids with short distances of 2.21 and 2.37 Å, respectively, thereby suggesting the existence of van der Waals interactions to maintain the protein conformation. In contrast, no polar contact was identified in the mutation of Y16N, K17R, and A18V, whereas K15R and Q19T had polar contacts with Lys6, Tyr3, and Ser20. The change in interactions from charge matching of certain side groups and van der Waals coordination between these mutation regions may result in the inability to produce antifungal proteins in *E. coli* (Liu et al., 2020). Although the ability of this conserved chitin-binding domain to inhibit fungi cannot be demonstrated, the motif would affect whether or not the rAFP can be expressed and folded.

The rAFP had an antifungal effect on *P. variotii* CICC40716. Meanwhile, no cytotoxicity was observed in the RAW264.7 cell. The selective advantage suggested that the rAFP had the potential as a fungicide for medicine. Little attention has been devoted to the thermostability of AFP from *A. giganteus*. However, a volume of research has been published on thermostable plant defensins from cowpea seeds and *Phaseolus vulgaris* L. (Chan and Ng, 2013; Thery and Arendt, 2018). In the present study, 100 μg/ml of rAFP still retained over 40% antifungal activity after 50°C pretreatment for 5 h, thereby suggesting the certain thermal stability of the rAFP (Figure 4). Furthermore, its thermostability may be due to high β-sheet contents, and salt-bridge interactions of Lys, Asp, Arg, and Glu account for 28.8% of the rAFP (Zhou et al., 2008; Niu et al., 2015). Although tryptophan was responsible as a good fluorescent indicator in the change in the molecular structure because of its high sensitivity to photophysical parameters (Zhdanova et al., 2015), tyrosine could be used to monitor the conformational change in tryptophan-lacking protein (Zhdanova et al., 2017). In this study, the intrinsic fluorophores of tyrosine and phenylalanine of the rAFP distributed around the cysteine disulfide bonds were observed under a maximal emission peak at 343 nm (Figure 6). As the heat treatment time of the rAFP increased, the fluorescence emission intensity declined and shifted from 343 to 335 nm, thereby indicating the conformational change in the protein structure. This result was similar to the tryptophan as the fluorescent probe to measure protein unfolding, ranging from 330 nm (hydrophobic environment) to 350 nm (hydrophilic environment) of the fluorescence peak (Kotov et al., 2021). Therefore, CD spectroscopy was used to further investigate the effect of thermal unfolding on the secondary structure of the rAFP. The main secondary structure of the antiparallel arrangement in the rAFP was consistent with the protein structure of the previous report (Table 1) (Campos-Olivas et al., 1995). Different protein structures, such as the increased helix and β-sheet contents, additional salt bridges, decreased cavities, and chain flexibility, could be responsible for the contribution of thermostability (Zhou et al., 2008). CD spectroscopy revealed that the helical and β-turn structures of the rAFP attributed to thermal stability collapsed as the heating time increased. This result was consistent with the findings of antifungal activity and fluorescence.

Several efflux transporters in fungi including ATP binding cassette (ABC) superfamily transporters, major facilitator superfamily (MFS) transporters, multidrug and toxic compound extrusion (MATE), small multidrug resistance (SMR), and resistance-nodulation-cell division (RND) are correlated with resistance to fungicides (Kuroda and Tsuchiya, 2009; Rahman et al., 2017; Samaras et al., 2020). In general, these efflux transporters associated with multidrug resistance (MDR) are expressed against fungicides such as *Penicillium digitatum* and *Zymoseptoria tritici* (Omrane et al., 2015; Wu et al., 2016). A total of 19 efflux transporters were identified in this study in the DEGs of *P. variotii* CICC40716 corresponding to 11 and eight upregulated and downregulated genes by RNA-seq analysis, respectively (Table 2). A limited number of ABC transporters and MATE that contributed to upregulation was perceived. In contrast, the gene expression levels of MFS transporters were upregulated and downregulated. Thus, the ABC transporters and MATE served as the sensitive gene induced by the rAFP.

The cell wall protects the cell from osmotic change and shapes the cell form, and several carbohydrate metabolic activities are necessary for cell wall biosynthesis (Song et al., 2022). Mitogen-activated protein kinase (MAPK) is necessary for cell wall integrity pathway (CWIP) activation (Rocha et al., 2015; Gurgel et al., 2019). In this study, the changes in the metabolic pathways of downregulation such as amino sugar and nucleotide sugar metabolism, as well as the MAPK signaling pathway involved in the cell wall integrity, can be found after mapping the DEGs to the KEGG database (Figure 11). The result corresponded to the PI assay, thereby suggesting the collapse of the cell wall and damage to the cell membrane (Figure 7). In contrast, tyrosine metabolism in the upregulation pathway was greatly affected, which may be responsible for drug resistance (Zhang et al., 2021). This was also observed in the extract of *Amaranthus tricolor* leaf as a bacteriostatic agent against *Acidovorax avenae* subsp. *citrulli* (Zhang et al., 2021).

ROS increase induced by antifungal protein is one of the factors that can inhibit fungal growth (Oshiro et al., 2019). Thus, the antioxidant defense system was triggered to eliminate ROS by activating antioxidant enzymes such as catalase, peroxidase, and superoxide dismutase (Glorieux and Calderon, 2017). In the present study, the corresponding upregulated DEGs were enriched in response to the oxidative stress of biological processes based on the GO database (Figure 12). Catalase and cytochrome c peroxidase were identified and upregulated in response to oxidative stress after the rAFP treatment of *P. variotii* CICC40716 (Glorieux and Calderon, 2017; Shin et al., 2017). Moreover, the corresponding upregulated DEGs were enriched in the oxidoreductase activity of molecular function. The encoding proteins of laccase, multicopper oxidase, and nitroreductase contributing to ROS scavenging could be found (Pompeu et al., 2019; Zhou et al., 2021; Monteiro et al., 2022). These results suggested that the ROS increase in the *P. variotii* CICC40716 treated by the rAFP induced the activation of antioxidant defense enzymes.

## 5. Conclusion

IFIs with drug resistance are an issue of global public health. Thus, new antifungal drugs are necessary to be developed. Herein, the rAFP derived from the optimal sequence of *A. giganteus* (CAA43181) was expressed in *E. coli*, and it was capable of antifungal effects against the *P. variotii* CICC40716. After pre-heating for 8 h at 50°C, the great fluorescence emission of the rAFP was shifted from 343 to 335 nm. Moreover, the helix and β-turn of the rAFP decreased with the increase in thermal pretreatment, indicating the destruction of the protein secondary structure. However, the rAFP was not cytotoxic to RAW264.7 cells and may influence the integrity of the cell wall and cell membrane of the *P. variotii* CICC40716. Furthermore, the upregulation of the antioxidant defense system indicated the increase in ROS, thereby resulting in fungal death. Consequently, the rAFP can be a fungal inhibitor for medical treatment.

## Data availability statement

The datasets presented in this study can be found in online repositories. The names of the repository/repositories and accession number(s) can be found in the article/Supplementary material.

## Author contributions

Y-PC contributed to conceptualization, writing original draft preparation, writing review and editing, project administration, and funding acquisition. YL, FC, and HW contributed to data curation. SZ contributed to the software. All authors contributed to the manuscript and approved the submitted version.
