# Supplementary material for: Characterization and expression of fungal defensin in Escherichia coli and its antifungal mechanism by RNA-seq analysis
Source: Front Microbiol. 2023 Jun 14;14:1172257. doi: 10.3389/fmicb.2023.1172257 (PMC10306309; doi:10.3389/fmicb.2023.1172257)
Supplement: Supplementary file 1 [file Data_Sheet_1.DOCX]

Supplementary Material

Characterization and overexpression of optimal fungal defensin in *Escherichia coli* and its antifungal mechanism by RNA-seq analysis

Yu-Pei Chen^123,*^, Yingying Li^2,4^, Fangfang Chen^12^, Hongtan Wu^12^, Shudi Zhang^12^

*** Correspondence:** Corresponding Author: 201600080006@xmmc.edu.cn


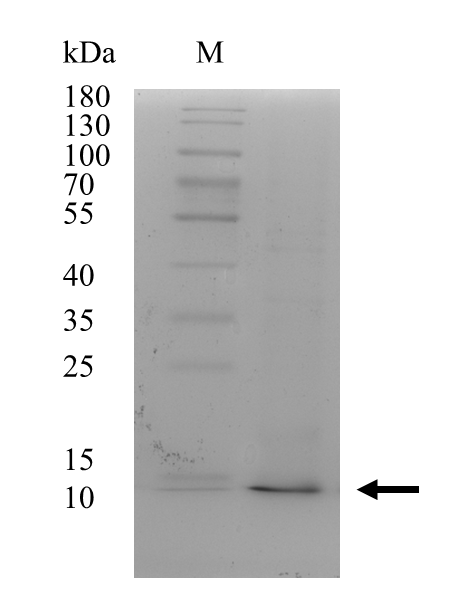


**Supplementary Figure 1.** The rAFP purification using nickel column. The arrow indicates the purified protein.


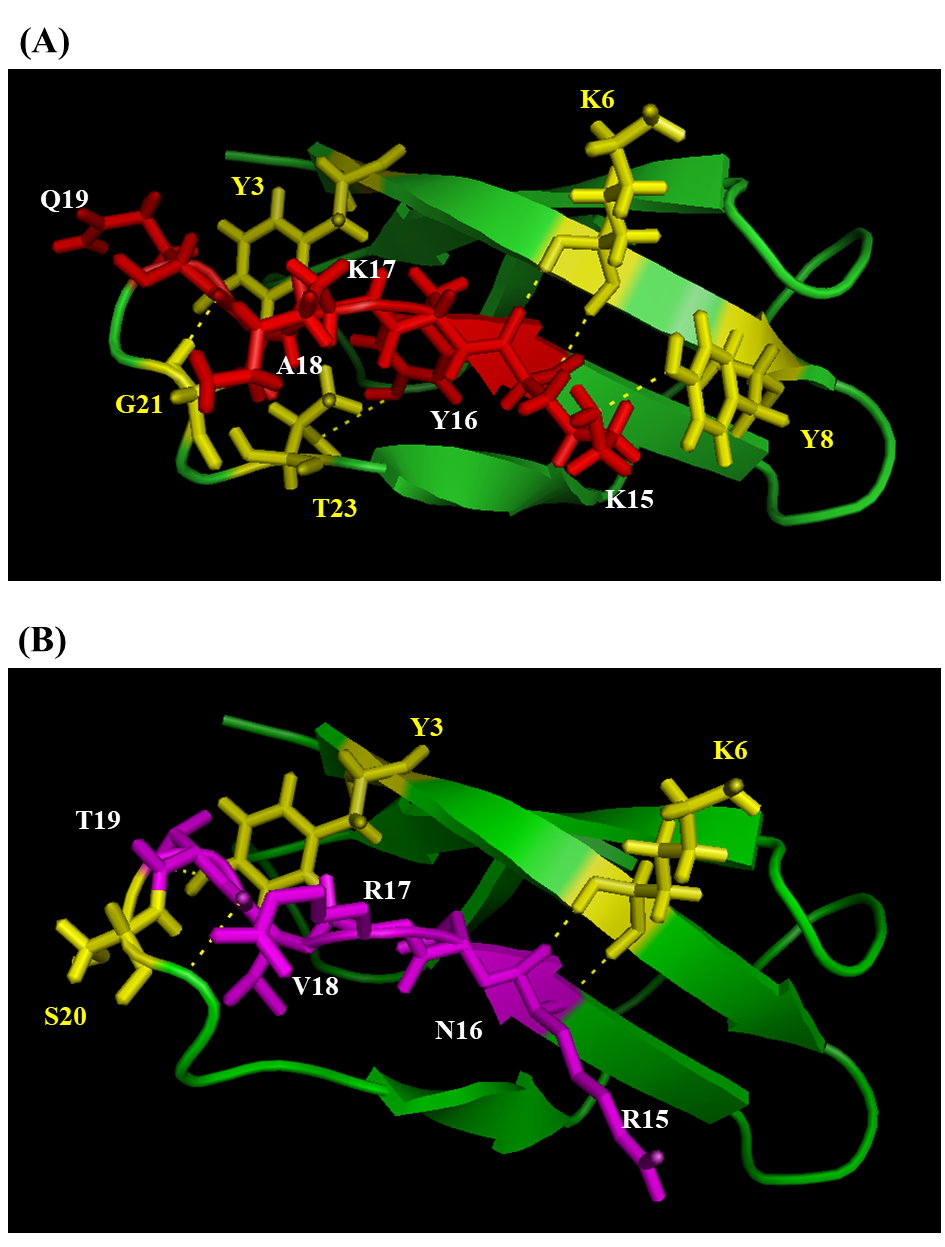


**Supplementary Figure 2.** Protein structure prediction of the (A) rAFP and (B) its mutation of the chitin-binding motif according to the PDB database (1AFP). The protein structures were implemented in PyMol ([www.pymol.org](http://www.pymol.org)). The yellow dotted line indicates the polar contact.

**Supplementary** **Table 1.** The mature sequences of antifungal genes were designed using OptimumGene software.

| Antifungal genes | Optimized Mature DNA sequences^a^ |
| --- | --- |
| *Fusarium poae*  (CAR79017) | CTGGAGTACTGGGGCAAGTGCACCAAAGCGGAAAACCGTTGCAAGTATAAAAACGACAAGGGCAAAGATGTGCTGCAGAACTGCCCGAAATTCGACAACAAGAAATGCACCAAGGATGGTAACAGCTGCAAATGGGACAGCGCGAGCAAGGCGCTGCCGTGCTAC |
| *Neosartorya fischeri*  (CAQ42994) | CTGGAGTACAAGGGTGAATGCTTCACCAAAGACAACACCTGCAAGTACAAAATCGATGGCAAGACCTATCTGGCGAAATGCCCGAGCGCGGCGAACACCAAGTGCGAGAAAGACGGTAACAAGTGCACCTACGATAGCTATAACCGTAAGGTGAAATGCGACTTTCGTCAC |
| *Aspergillus giganteus*  (CAA43181) | GCGACCTACAACGGCAAGTGCTATAAGAAAGACAACATCTGCAAGTACAAAGCGCAGAGCGGCAAGACCGCGATTTGCAAATGCTATGTGAAGAAATGCCCGCGTGACGGTGCGAAATGCGAGTTCGATAGCTACAAGGGCAAATGCTATTGC |
| *Fusarium asiaticum*  (CAR79023) | CTGGAGTACTGGGGCAAGTGCACCAAAGCGGAAAACCGTTGCAAGTATAAAAACGACAAAGGCCGTGATGTGCTGCAGAACTGCCCGAAGTTCGACAACAAGAAATGCACCAAGGATGGTAACAGCTGCAAATGGGACAGCGCGAGCAAGGCGCTGACCTGCTAC |
| *Aspergillus clavatus*  (ABR10398) | GCGCGTGACGAGAGCGCGGTGCAGGCGACCTACGACGGCAAGTGCTACAAGAAAGATAACATCTGCAAGTACAAGGCGCAAAGCGGCAAAACCGCGATTTGCAAGTGCTATGTGAAAGTTTGCCCGCGTGACGGTGCGAAGTGCGAGTTCGATAGCTACAAGGGCAAATGCTATTGC |
| *Colletotrichum gloeosporioides*  (ELA33717) | TTCATCACCTACACCGGCAAGTGCACCCGTGGCCGTAACTATAAAGAGGACACCTGCAAGTTTAAGGGTCAGAAGGGCAAAACCACCATTGTGCGTTGCCCGCGTTTCGCGAACCAACGTGTTAGCTTTGAT |
| *Penicillium chrysogenum*  (DOEXD3) | CTGAGCAAGTTCGGTGGCGAGTGCAGCCTGAAACACAACACCTGCACCTACCTGAAGGGTGGCAAAAACCACGTGGTTAACTGCGGTAGCGCGGCGAACAAGAAATGCAAGAGCGACCGTCACCACTGCGAGTATGATGAACACCACAAACGTGTGGACTGCCAGACCCCGGTT |
| *Fusarium boothii*  (CAR79010) | CTGGAGTACTGGGGCAAGTGCACCAAAGCGGAAAACCGTTGCAAGTATAAAAACGACAAGGGCAAAGATGTGCTGCAGAACTGCCCGAAATTCGACAACAAGAAATGCACCAAGGATGGTAACAGCTGCAAATGGGACAGCGCGAGCAAGGCGCTGACCTGCTAC |

^a^ The underline represents the optimized codon sequences.

**Supplementary** **Table 2.** The summary of RNA-seq data performed by an Illumina NovaSeq 6000.

| Sample | Clean read | Base number | GC content | % ≧ Q30^a^ | Assembled transcripts | Ratio of assembled transcripts |
| --- | --- | --- | --- | --- | --- | --- |
| Control-1 | 20,112,683 | 6,018,360,278 | 54.27% | 92.01% | 18,689,798 | 92.93% |
| Control-2 | 21,418,501 | 6,409,599,944 | 53.97% | 93.83% | 19,768,136 | 92.29% |
| Control-3 | 21,210,992 | 6,351,925,594 | 54.37% | 94.01% | 19,687,427 | 92.82% |
| rAFP-1 | 21,293,117 | 6,375,161,722 | 54.14% | 94.15% | 19,680,605 | 92.43% |
| rAFP-2 | 23,178,493 | 6,937,147,412 | 54.19% | 93.98% | 21,425,944 | 92.44% |
| rAFP-3 | 24,680,016 | 7,388,224,032 | 54.20% | 93.83% | 22,670,326 | 91.86% |

^a^ %≥Q30: percentage of bases with Q-score no less than Q30.

**Supplementary Table 3.** The 15 most up-regulated genes responding to the ratio of the rAFP treatment to control in *P. variotii* CICC40716.

| No. | ID | DNA size (bp) | NR annotation | COG class annotation | log_2_ ratio |
| --- | --- | --- | --- | --- | --- |
| 1 | c20493.graph_c1 | 8259 | Predicted protein | -- | 8.127 |
| 2 | c24025.graph_c1 | 12142 | WD repeat protein | Posttranslational modification, protein turnover, chaperones | 7.791 |
| 3 | c13322.graph_c0 | 2323 | Dihydrouridine synthase family protein, putative | Translation, ribosomal structure and biogenesis | 6.146 |
| 4 | c20567.graph_c1 | 3214 | Sodium/phosphate symporter | Inorganic ion transport and metabolism | 6.063 |
| 5 | c13492.graph_c0 | 3198 | MFS transporter, putative | Carbohydrate transport and metabolism | 5.633 |
| 6 | c13215.graph_c0 | 504 | G-patch domain protein (Spp2), putative | -- | 5.005 |
| 7 | c22577.graph_c0 | 1845 | Cytochrome P450 | -- | 4.980 |
| 8 | c20142.graph_c0 | 1446 | Hypothetical protein | Energy production and conversion | 4.850 |
| 9 | c20865.graph_c0 | 8426 | Quinate pathway repressor protein QutR | Amino acid transport and metabolism | 4.798 |
| 10 | c19547.graph_c0 | 2769 | FF domain protein | -- | 4.783 |
| 11 | c24625.graph_c0 | 1549 | Conserved hypothetical protein | -- | 4.577 |
| 12 | c24551.graph_c0 | 1270 | IgE-binding protein | -- | 4.577 |
| 13 | c22716.graph_c1 | 5010 | DNA replication licensing factor Mcm6, putative | Replication, recombination and repair | 4.516 |
| 14 | c21456.graph_c1 | 2500 | Hypothetical protein | Inorganic ion transport and metabolism | 4.466 |
| 15 | c18369.graph_c0 | 4427 | Potassium/sodium efflux P-type ATPase, fungal-type, putative | Inorganic ion transport and metabolism | 4.453 |

**Supplementary** **Table 4.** The 15 most down-regulated genes responding to the ratio of the rAFP treatment to control in *P. variotii* CICC40716.

| No. | ID | DNA size (bp) | NR annotation | COG class annotation | log2 ratio |
| --- | --- | --- | --- | --- | --- |
| 1 | c24256.graph_c1 | 19529 | IQ and HECT domain protein | Inorganic ion transport and metabolism | -6.633 |
| 2 | c24077.graph_c0 | 10496 | Aminopeptidase | Amino acid transport and metabolism | -6.522 |
| 3 | c13297.graph_c0 | 1630 | Hypothetical protein | General function prediction only | -6.489 |
| 4 | c24160.graph_c1 | 9423 | Hypothetical protein | Carbohydrate transport and metabolism | -5.385 |
| 5 | c13231.graph_c0 | 1286 | Cyanide hydratase/nitrilase, putative | General function prediction only | -5.382 |
| 6 | c15392.graph_c0 | 424 | Hypothetical protein | -- | -4.981 |
| 7 | c24458.graph_c3 | 1874 | Secretion-associated GTP-binding protein sarA | -- | -4.945 |
| 8 | c18406.graph_c0 | 1522 | Hypothetical protein | Coenzyme transport and metabolism | -4.648 |
| 9 | c21938.graph_c0 | 3243 | Hypothetical protein | -- | -4.440 |
| 10 | c17822.graph_c0 | 891 | Conserved hypothetical protein | General function prediction only | -4.414 |
| 11 | c24091.graph_c1 | 7208 | Conserved hypothetical protein | -- | -4.350 |
| 12 | c21691.graph_c1 | 1730 | Nuclear migration protein (ApsA), putative | Coenzyme transport and metabolism | -4.246 |
| 13 | c21594.graph_c1 | 1690 | Hypothetical protein | -- | -4.148 |
| 14 | c18457.graph_c0 | 2236 | Diacylglycerol pyrophosphate phosphatase | -- | -3.816 |
| 15 | c20455.graph_c0 | 1222 | Predicted protein | Carbohydrate transport and metabolism | -3.813 |

**Supplementary** **Table 5.** The comparison of gene expression including up-regulation and down-regulation by RNA-seq and real-time PCR.

| ID | Annotation | log_2_ ratio by RNA-seq | Fold change by real-time PCR |
| --- | --- | --- | --- |
| c24077.graph_c0 | Aminopeptidase | -6.522 | -2.103 |
| c24160.graph_c1 | Hypothetical protein | -5.385 | -1.402 |
| c24458.graph_c3 | Secretion-associated GTP-binding protein sarA | -4.945 | -2.713 |
| c21938.graph_c0 | Hypothetical protein | -4.440 | -48.911 |
| c20865.graph_c0 | Quinate pathway repressor protein QutR | 4.798 | 3.027 |
| c22577.graph_c0 | Cytochrome P450 | 4.980 | 8.113 |
| c13492.graph_c0 | MFS transporter | 5.633 | 1.524 |
| c13322.graph_c0 | Dihydrouridine synthase family protein | 6.146 | 1.873 |
